# Fracture of the shoulder girdle in multiply injured patients - an imperative for a high level of suspicion for associated neurovascular injuries

**DOI:** 10.1186/1754-9493-7-24

**Published:** 2013-07-07

**Authors:** Senat Krasnici, Jörg Schmidt, Kolja Reimann, Wolfgang Ertel, Tobias Topp

**Affiliations:** 1 Department of Orthopaedic and Reconstructive Surgery, Charité University Medicine Berlin, Campus Benjamin Franklin, Hindenburgdamm 30, 12003 Berlin, Germany

**Keywords:** Clavicle fractures, Scaphulothoracic dissociation, Multiply-injured patients, Associated injuries, Brachial plexus, Subclavian artery

## Abstract

**Background:**

The combination of a bony injury to the shoulder girdle and damage to the brachial plexus and the subclavian vessels is a rare finding. The cases of this combined injury pattern described in the literature are most notably reported in multiply-injured patients after high velocity trauma.

**Findings:**

Three cases were admitted to our hospital after motorcycle accidents resulting in a combination of severe bony injuries to the shoulder girdle, to the subclavian artery and a lesion to the brachial plexus. Based on these three clinical cases the patterns of injury, as well as primary and secondary treatment approaches are presented.

**Conclusion:**

The early detection of these injuries can be difficult in given acute, life threatening injuries addressed first in these multiply injured patients. A high level of suspicion, in conjunction with standardized ATLS based institutional protocols for secondary and tertiary survey, should increase the likelihood of a timely detection and early management of these rare but potentially devastating injuries.

## Background

Fractures of the shoulder girdle accompanied with vascular and plexus injuries are infrequent but can have a potentially devastating outcome. Injuries to the brachial plexus are either caused by distraction injuries as seen in scaphulothoracic injuries or direct injuries to the trunks, cords and nerves. Injuries to the brachial plexus occur in approximately 5% of polytrauma patients involved in motorcycle crashes [1]. Distraction injuries with definite and unrecoverable neurological deficits are caused by root avulsions, where the rootlets are torn out of the spinal cord. In contrast postganglionic stretch injuries or ruptures show a better prognosis [2,3]. Brachial plexus injuries are associated with a 10%–25% incidence of arterial injury [2]. These injuries may lead to life threatening bleedings in multiply injured patients and cause hemodynamic instability. Both, the detection and treatment of an acute arterial injury as well as the treatment of plexus injuries have to be integrated in treatment algorithms for polytrauma patients. Over a period of six months, three patients were admitted to our hospital after motorcycle accidents resulting in a combination of injuries to the bony shoulder girdle, the subclavian artery and brachial plexus. Based on these three clinical cases the injury patterns, as well as treatment approaches are presented.

## Case presentation

### Case I

**Figure 1:**
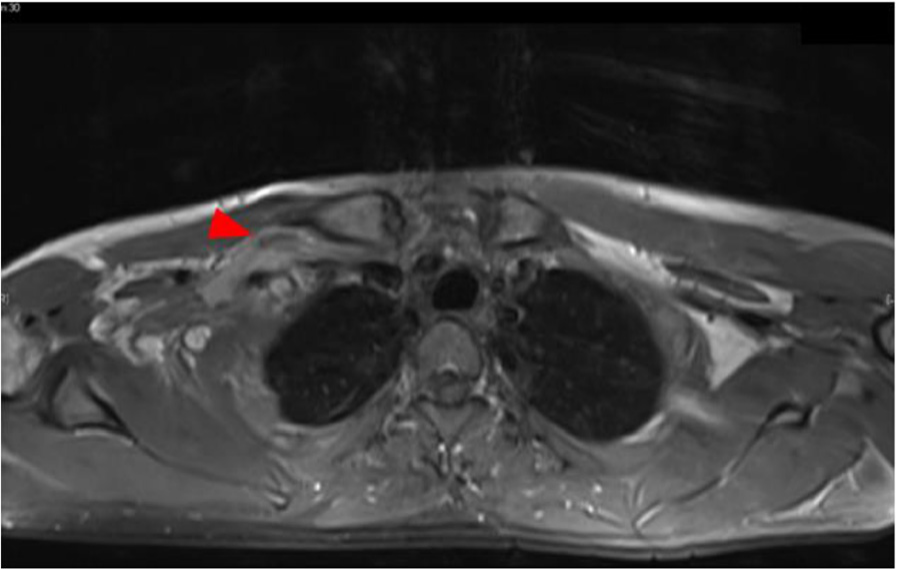
**MRI of infraclavicular level.** Abnormal exposure of the right brachial plexus, indicating a partial injury.

A multiply-injured (ISS 35) seventeen-year-old man who was involved to a motorcycle accident was admitted to our emergency department after in the field intubation. The initial trauma management in our institution was performed according to ATLS guidelines with chest x-ray, pelvic a.-p.-view and a focused ultrasound assessment for trauma (FAST). Radiographs showed a displaced fracture of the left clavicle, a femoral fracture and a hemothorax with consequent hemodynamic deterioration. An emergency thoracotomy was performed to control haemorrhage. During the thoracotomy, several deep ruptures of the lung in segment 9 and 10 were identified as bleeding sources and could be stopped. No major vessels were injured, a clamping was not necessary. The femur fracture was stabilized by external fixation according to damage control rules in multiply-injured patients. After surgery, a motoric dysfunction and complete loss of sensation in the patients left arm was found. In the subsequently performed MRI scan a disruption of the roots of the brachial plexus was seen (Figure 1). The definitive osteosynthesis of the femoral and clavicle fractures was performed 8 days after trauma. One month after trauma the patient was transferred to a neurosurgical clinic for further treatment. In two steps, extra-plexular neurotizations, transferring spinal accessory nerve to the suprascapular nerve and transferring the intercostal nerves III and IV to the musculocutaneous nerve were performed. The patient was followed up 18 months after trauma. After this period the clavicle and the femoral fracture healed and a removal of the intramedullary femur nail was performed. An active abduction of the arm up to 30° could be reached. The muscle strength of the biceps was M2 (contraction with gravity eliminated) using the British Medical Research Council Grading System (M0-5).

### Case II

A thirty one-year-old man (ISS 41) was admitted to our emergency department following a motorcycle accident. On admission he was alert and oriented. Primary survey yielded tachycardia and a massive hematoma of the left shoulder. Focused examination of the left upper limb revealed paralysis of the entire arm with the radial pulse neither being palpable nor traceable by Doppler ultrasound. Chest x-ray showed a fracture of the left clavicle. The patient was emergently taken to the operating room, where the suspicion of a ruptured subclavian artery was confirmed. A vessel repair using an interpositional PTFE graft between the subclavian and the axillary artery was performed. In addition a complete brachial plexus avulsion was found. The displaced fracture of the clavicle was simultaneously fixed by plating. Femoral fractures on both sides and a tibial fracture on the left side were treated by external fixation. Definitive osteosynthesis was done 10 days after trauma. In addition, the CT scan performed after emergency surgery showed a subdural haematoma, and a pneumothorax. The patient was transferred to a neurosurgical clinic 7 weeks after trauma for the reconstruction of the plexus injury. A neurotization from the spinal accessory nerve to the musculocunaeous nerve using a suralis nerve graft and a neurotization using the phrenic nerve to the musculocutaneous nerve was performed. The last follow up was 1 year after trauma, the patient still showed a complete loss of function of the left arm. The strength of the biceps muscle was only M1 (fasciculations observed in the muscle).

### Case III

**Figure 2:**
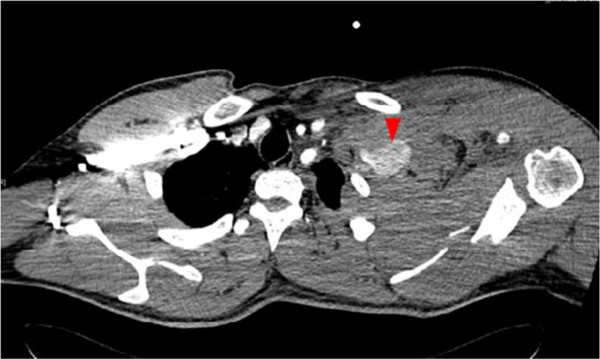
**Polytrauma CT scan.** Extravasation of contrast agent in left subclavian artery indicating rupture.

**Figure 3:**
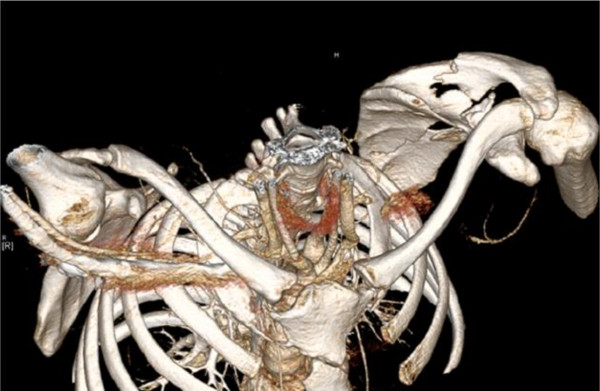
**CT-3D-reconstruction of shoulder girdle.** Visualization of displaced scapula fracture on the left side. Note also the extravasation of contrast agent and the missing presentment of the ipsilateral subclavian artery.

**Figure 4:**
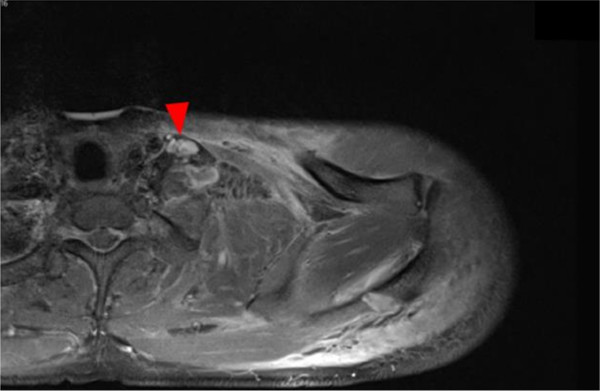
**MRI of left shoulder girdle.** Tear of the left brachial plexus.

A thirty two-year-old male (ISS 43) who suffered a motorcycle accident, primary trauma survey yielded a pulmonary contusion and left scapular body fracture. Immediate intubation of the patient in the emergency room due to respiratory insufficiency was performed. Subsequent CT scan confirmed a massive contusion of the lung and revealed an active extravasation of contrast agent at the level of the left shoulder (Figures 2 and 3). Clinically, pulselessness of the left arm was realized whilst the hemodynamic condition had deteriorated. An emergency vascular intervention was performed to bypass the ruptured subclavian artery to the brachial artery using a venous graft. A rupture of the plexus could not be detected. Postoperatively, the conscious patient perceived a complete palsy of the left arm. MRI scans showed no preganglionic lesions but postganlionic fraying from C6 to C8 and a complete rupture of all truncs with a surrounding edema (Figure 4). Electromyography showed a severe axonal lesion of the whole plexus with a complete loss of function. The scapular fracture was treated by conservative immobilization. Six weeks after the accident the patient was admitted to a neurosurgical clinic for further treatment. A neurotization of the musculocutaneous nerve using the intercostal nerves III and IV was performed. In addition the intercostal nerves V and VI were transferred to the forearm using the superficial branch of the radial nerve as a graft. This was done to prepare a gracilis transfer in case of sufficient innervation of the graft. The patient was lost to follow up.

## Discussion

Injuries of the brachial plexus and subclavian vessels can be associated with bony injuries of the shoulder girdle. They are found in approximately 5% of polytrauma patients suffering a motorcycle accident [1]. Zelle et al. published a study presenting long term outcomes after scapulothoracic dissociations. 25 patients were included to the study over a period of 24 years. Poor functional outcome after complete plexus lesions was found [4]. Even with modern reconstruction techniques providing far greater restoration than was possible a few years ago, sequelae such as persistent neurological deficits or functional loss of the entire arm and even unbearable neuropathic pain can cause patients to request for amputation.

Acute plexus injuries originate from a torsion-distraction-like movement to the upper extremity in which the head and neck distends away from the ipsilateral shoulder or from hyperextension-distraction injuries of the upper limb, as seen in the three high velocity motorcycle accident cases presented above. Rupture or incomplete tear of subclavian vessels can account for hemodynamic instability, along with bleeding into large body cavities. In sedated or unconscious patients, the inability to adequately examine the nervous system of the upper extremity challenges the initial evaluation. To detect and treat these life-threatening injuries during the primary trauma survey, strict adherence to ATLS based algorithms is essential. As presented in case I and II, in hemodynamic unstable patients, emergent surgical treatment to establish haemostasis may precede a trauma CT scan. In Case II and III the injured extremity was pulseless. Patients with severe extremity trauma and suspected arterial injury should undergo immediate surgery if hard signs like: pulsatile bleeding, expanding hematoma, palpable thrill and audible bruit are present [5,6]. Literature validated the use of ankle brachial or arterial pulse indexes (ABI and API respectively), which were shown to reliably detect arterial injury to a limb and are easily performed in the Emergency room [5,7]. In hemodynamic stable patients (Case III) a CT scan (including ateriography) can be indicated as part of the primary survey th detect the exact location of the bleeding.

**Figure 5:**
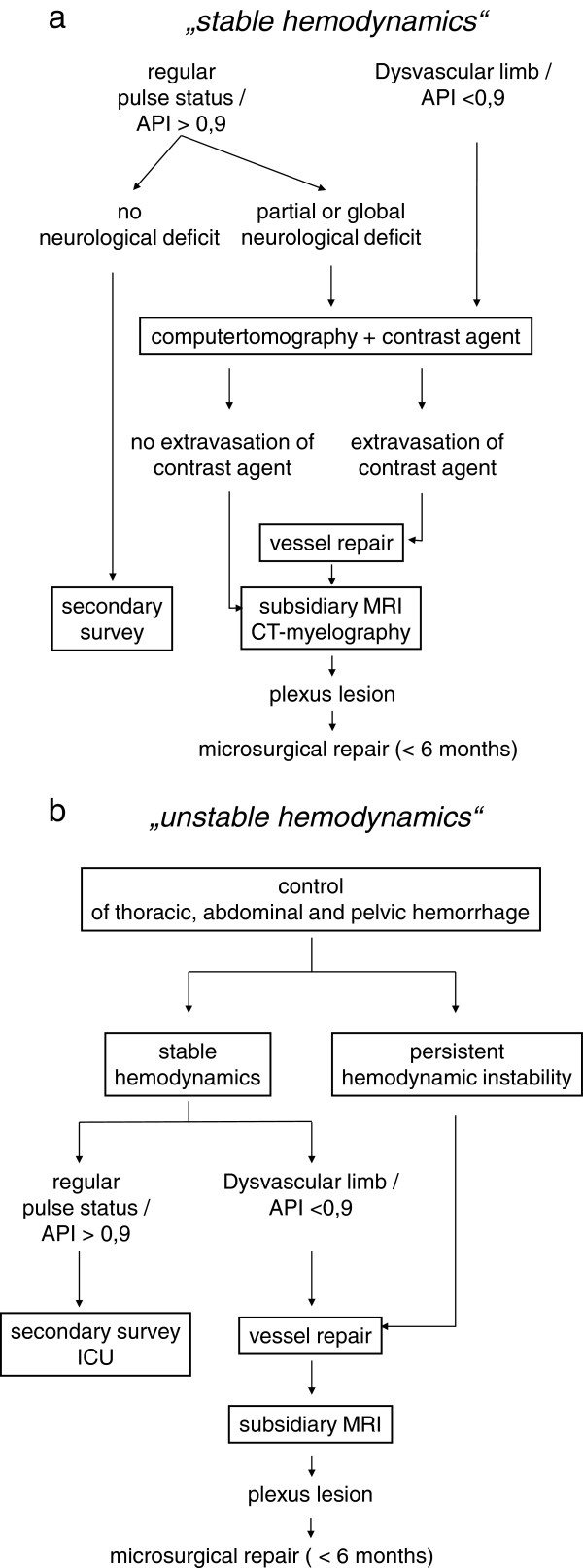
**Algorithm for the management of injuries to the subclavian vessels and brachial plexus in multiply-injured patients in hemodynamically stable (a) and unstable (b) condition.** (API = Arterial Pressure Index).

Further diagnostics such as MRI scans can be performed during secondary survey in the hemodynamic stable patient. Once a neurological deficit of the upper extremity is detected, an MRI scan is indicated, even if the lesion of the brachial plexus has been defined within the context of surgery, e.g. for an active bleeding. While clinical examination is the gold standard, the knowledge of the extend and the exact localization of the lesion as seen in MRI imaging can aid in the concerted planning of further treatment options. The current gold standard imaging for evaluation of root-level injuries is computed tomography with myelography [2]. The development of a pseudomeningoceles, 3–4 weeks after injury highly indicates a root avulsion [2,8]. There is no general agreement on the timing for treatment of injuries of the plexus. Penetrating injuries are indications for immediate or early exploration once the patient is stable and, if possible, direct end-to-end repair. The early functional reconstruction in the first months is recommended when there is a high suspicion of root avulsion [9]. Given the chance in treatment protocols in the last decade in favour of local nerve transfers many authors recommend this approach in root avulsions if transfer options remain, when the patient has recovered from the early trauma aftermath. These nerve transfers are done outside the zone of injury and tedious dissection of scarred plexal anatomy can be avoided. This is in contrast to direct reconstruction of postganglionic plexus injuries, where one needs to explore the injured region directly. Here an early surgical exploration before the development of scar and neuroma may allow an easier exploration and identification if injured structures [2,9]. In multiple injured patients with concomitant severe injuries an earlier exploration or reconstruction is often not possible. In these cases surgical exploration is recommended 3–6 months after trauma, if no signs of reinnervation are found in clinical exam and electroneurographic studies [2]. Figure 5 provides an algorithm for the management of these injuries in multiply-injured patients with stable (a) and unstable hemodynamics (b) (Figure 5).

## Conclusion

In high speed complex injuries of the shoulder girdle, one must always suspect and actively rule out combined injuries to the brachial plexus and the axillary or subclavian vessels.

## Competing interests

The authors declare that they have no competing interests.

## Authors’ contributions

SK conceived the study, drafted and reviewed the manuscript. JS participated in study design, coordination and manuscript drafting. KR participated in data aquisition. TT was involved in drafting and revising the manuscript and gave final approval together with WE. All authors read and approved the final manuscript.
